# Diversity, distribution, agronomic and post-harvest management of local barley (*Hordeum vulgare* L.) variety in South Wollo, Ethiopia

**DOI:** 10.1371/journal.pone.0250899

**Published:** 2021-05-06

**Authors:** Akale Assamere, Alemu Mamuye, Kassahun Amare, Mulugeta Fiseha

**Affiliations:** 1 Department of Biology, Mekdela Amba University, South Wollo, Ethiopia; 2 Department of Plant Science, Mekdela Amba University, South Wollo, Ethiopia; 3 Department of Natural Resource Management, Mekdela Amba University, South Wollo, Ethiopia; Feroze Gandhi Degree College, INDIA

## Abstract

The structure of barley varieties were studied, using structured and semi-structured queries, at Legambo, Tenta and Worailu districts of South Wollo, Ethiopia. Eight local barley varieties (Belg, Ginbot, Sene/Nech, Tikur, Holker, Traveler Tegadime and Temezhi) were identified, and got their names found on seed color and planting season. According to farmers, Tegadime is the production leader among all, but the source of seeds and the market chain are the limiting factors for its popularity and this is why it’s not famous is because of the low price of the seed. Thus, Sene/Nech found to be popular and shared 46.91% at Tenta, 48.47% at Legambo and 51.55% at Wereilu followed by Tikur and Ginbote. High barley diversity was noted at Tenta (E = 0.773) followed by Wereilu (E = 0.678) and Legambo (E = 0.606). Sene/Nech (0.67), Belg (0.62), Tegadime (0.59), Tikur (0.55) and Ginbote (0.54) were found to be shared, but Traveler, Holker and Temezhi were rarely found. At farm, most farmers were plowing twice before sowing using horse. Biological fertilizer usages were well practice at Tenta, Legambo and Were’ilu, respectively. While, inorganic fertilizer usage was better at Wereilu, but none at Legambo. Pest management was better at Wereilu and hand weeding is a common system, but low at Legambo, and mowing by sickle, threshing by horse and store in Gotera were a shared practice. Farmers use outdated tools for agricultural practice and the yield is losing due to unavailable of update machinery. So, different managing approaches and new harvesting technologies should address.

## Introduction

In the developed countries, barley is mainly used for animal feed, malting and brewing. However, in the developing countries including Ethiopia, barley is producing mostly for food [1] and it has a long history in cultivation at Ethiopian highlands [2]. Barley (*Hordeum vulgare* L.) is the most important small grain in Ethiopia after Wheat, Teff and Maize [3].

The major barley producing regions in Ethiopia are Oromia, Amhara, Tigray and southern nation nationality and Regional State, and they accounted for about 99.94% of the total national barley production [4]. The local varieties are could be more locally adapted and genetically variable than the modern cultivars [1]. It growing best at higher altitudes with an optimum ranges of 2000–3500 meters above sea levels [5]. Barley is grown as a ’meher’ (main season) crop at the higher elevation of Dega regions and also widely cultivating as a ’belg’ crop in many areas. It is grown mainly in Arssi, Bale, Shoa, Welo, Gojam and Gonder, Ethiopia [3]. In Ethiopia, it is produced by the subsistence farmers, mostly growing as local varieties with either little utilization or no application of fertilizers, pesticides and herbicides.

The local barley varieties are suffering serious genetic erosion [6] and on-farm genetic resource conservation system are gaining less attention. Similarly in this research area, recent status of on-farm local barley diversity, agronomic and post-harvest management practice is unknown or has not been documented. In this regards, a considerable assessment were conducted on the diversity, distribution, agronomic and post harvesting management in the three selected districts of South Wollo, Ethiopia. This is in fact that it creates a good insight for the figurative variation and structure of local barley varieties and tools incorporated in the agronomy. This info is also important for maintaining and future use of the varieties, and introducing updated globe technology and new machineries into the farmers [7].

## Materials and methods

Studies carried out in Legambo, Tenta and Werailu districts, South Wollo Zone Amhara Region, Ethiopia. Data collections were covered a total of 18 kebeles (6 kebeles in each district) and structured and semi-structured queries on were implemented to obtained the required information. One agricultural officer at each district and one expert at each kebele were selected for consultation purpose. We used 195 farmers for each Legambo and Were’ilu districts (33 farmers in each kebele) and 194 farmers for Tenta (32 farmers in each kebele) were used for a sampling. The sampling size was determined by Cochran (1977) technique and calculated as $n0=\frac{z^{2}pq}{e^{2}}$. Where, **n**_**0**_ is the sample size, **z** is the critical value of desired confidence level, **p** is the estimated proportion of an attributed that is present in the population, q = 1−p, **e** is the desired level of precision. Therefore, p = 0.5 and hence *q* = 1–0.5 = 0.5; *e* = 0.07; *z* = 1.96. So, n_0_ = (1.96)^2^(0.5)(0.5)/ (0.07)^2^ = 196. We used formula developed by Cochran (1977) with a precision level of ±7. $n=\frac{n0}{1+\frac{n0-1}{N}}$ Where, **N** designates total number of households in selected sub-districts/kebeles. Simpson’s diversity index [8] were done **(1- D) = 1∑(ni/Ni)**^**2**^. Where, **ni** represent individual of one particular local varieties, and **N** is sum of total number of individuals varieties. $Shannon\;diversity\;index\;also\;done:\;H\prime =-\sum_{i=1}^{s}PilnPi$. Where, **S** is number of varieties, and **Pi** is frequency of varieties i (ni/N), it means individuals of one particular species found (n) divided by the total number of individuals found (N). Evenness (E) was also calculated separately as a measure of the ratio of the observed diversity to the maximum diversity. It is defined by the function **E = H′/lnS**, Where, **H′** is Shannon index and **S** refers to the number of varieties recorded in each district. Beta (b) diversity [9] was also calculated using Sørenson’s similarity index. Sørenson’s similarity index = **2c/a+b**. Where, **a** represents number of varieties in district A, **b** is number of varieties in district B and **c** is number of local varieties common to both districts.

Name of the local barley varieties (improved, wild type), Maturity date (early, late), Farmers preference (concerning yield, market value, palatability), Seed morphology (two, six rowed, irregular types), and Seed and glume color (black, white, pink) were recorded. Data on agronomic practices like Land preparation (way of ploughing and frequency), Time of sowing, Types of fertilizer and way of application, and Crop protection system (weeding, herbicide and pesticide usage) were collected. Finally, postharvest management data were collected on way of mowing, threshing, and storing and types of storage. Descriptive statistics such as frequency distributions, means and percentage data were carried out by excel.

## Results and discussion

### Vernacular naming and distribution of local barley variety

Most local barley varieties got their names were founded on seed color, planting seasons and growth forms. Belg, Ginbote and Sene/Nech gebes has got their names based on planting season on March, May and June, respectively. This result was in line with [1] and revealed that Sene and Ginbote gebes has got its names according to the seasons of planting date of the seeds. Whereas, Nech gebes mean White barley and Tikur gebes mean Black barley and this indicate color of the seeds. Naming correlated with seed colors are in line with [10]. Barley, namely Holker and Traveler have got their names from the agricultural sectors, because it is improved varieties. However, the farmers in these districts are giving general names for these varieties and they entitled it Beera gebes. While, the name Tegadime means growing in good manner and got its name due to its height. All local barley possessed six rows, except Holker and Traveler which have two rows.

Eight local barley Varieties were documented, of that Sene/Nech, Tikur and Ginbote gebes were broadly grown (S1 Table and Table 2. Result in [1] showed that Nechita, Ginbote, Sene, Tikur gebes were the most common local varieties at North and South Wollo. Sene/Nech gebes was the most distributed at the three districts followed by Tikur gebes at Tenta and Legambo districts and Ginbote gebes at Wereilu district. Two improved varieties, namely Holker and Traveler were recorded in the districts and farmers use them for a trial, but both rarely found. 75% of the local barley varieties were six rowed and the remaining 25% were two rowed. Our result is not in line with [11], and 40.2% of two rowed, 37.7% of irregular and 22.2% of six rowed were documented in their results. Sene/Nech gebes found to be popular among many households (46.9% in Tenta, 48.47% in Legambo and 51.02% in Wereilu). Traveler, Holker, and Temezhi were cited by 13, four and eight farmers respectively and it showed rarely found in the districts. Eighteen local varieties were documented in North Western Ethiopia [10], but it showed poor distributions of local varieties among the three districts. In our study, low local barley variety richness (eight) was observed, but well distributed in all the studied districts.

### Farmer’s preference in regarding to barley production

In the insight of farmers, Tegadime is production leader followed by Tikur, Belg and Sene/Nech gebes. The most shared and popular local varieties were Sene/Nech gebes followed by Tikur and Belg (Table 2), but Nech gebes is lower in yield than Tegadime and Tikur (Table 1). Similar results were reported [10] in North Western Ethiopia. The price of Tegadime in the market is lower than others and due to its price farmers are not prefer it. Therefore, the distribution is limited. So, agroeconomic and marketing chain experts should be involved in the area to maintain the yield and speed up the price. Similarly [2] reported that usually farmers sow their own seed, less often seed exchange and loans were practiced.

**Table 1:** Respondent’s preferences of barley landraces and varieties based on productivity.

Local Barley Variety	R1	R2	R3	R4	R5	R6	R7	R8	Scored	Ranked
Sene/Nech	5	4	5	4	4	3	2	5	32	4^th^
Tegadime	1	1	2	2	1	2	3	1	13	1^st^
Temezhi	8	8	7	7	8	7	8	7	60	8^th^
Tikur	3	2	3	1	2	1	4	2	18	2^nd^
Belg	2	3	1	3	3	4	1	3	20	3^rd^
Ginbote	4	5	4	5	5	5	5	4	37	5^th^
Holker	6	7	8	6	6	7	6	6	52	6^th^
Traveler	7	6	6	8	7	6	7	8	55	7^th^

**Table 2:** Occurrence of local barley varieties in the three districts according to Simpson’s Index.

Local Barley variety	No of Farmer who cite the local barley			Cited farmers	Simpson index
	Tenta	Legambo	Wereilu		
Sene/Nech	91	95	100	286	0.67
Tegadime	13	3	12	28	0.59
Temezhi	7	1	0	8	0.22
Tikur	29	73	18	120	0.55
Belg	21	11	9	41	0.62
Ginbote	22	11	53	86	0.54
Holker	1	2	1	4	0.63
Traveler	10	0	3	13	0.36

### Determining the local barley diversity

High diversity indexes were recorded in Tenta (E = 0.773) followed by Wereilu (E = 0.678) and Legambo district (E = 0.606) (S1 Table). Approximately this was in agreed with [11], and in their result a 0.79 phenotypic diversity indexes were reported at Tigray, Northern Ethiopia. Shannon diversity index (H) showed high diversity in Tenta (H = 1.607) followed by Wereilu (H = 1.32) and Legambo (H = 1.179). In the simpson’s diversity index (D), Sene/Nech (0.67), Holker (0.63), Belg (0.62), Tegadime (0.59), Tikur (0.55), and Ginbote (0.54) were found to be common, and they are cited by 286, 4, 41, 28, 120 and 86 farmers, respectively (Table 2). Even if Holker was rarely found in the districts (cited by four farmers), it is evenly distributed (0.63) followed by Sene/Nech gebes (Table 2). This is why, even if the frequency is low, the Simpson’s diversity index is higher likewise [10]. However, Temezhi (0.22) and Traveler (0.36) were found only in the two districts and were cited by 8 and 13 farmers, respectively. This low Simpson’s diversity index revealed that the frequency of the local varieties were not distributed equally or restricted only in the two districts. Generally high similarity index were recorded in the association of Legambo with Tenta and Tenta with Wereilu (93.33% were alike). Similarly, 85.71% of resemblance was recorded between Legambo and Wereilu.

### Agronomic practice of local barley and fertilization utilization

**Fig 1:**
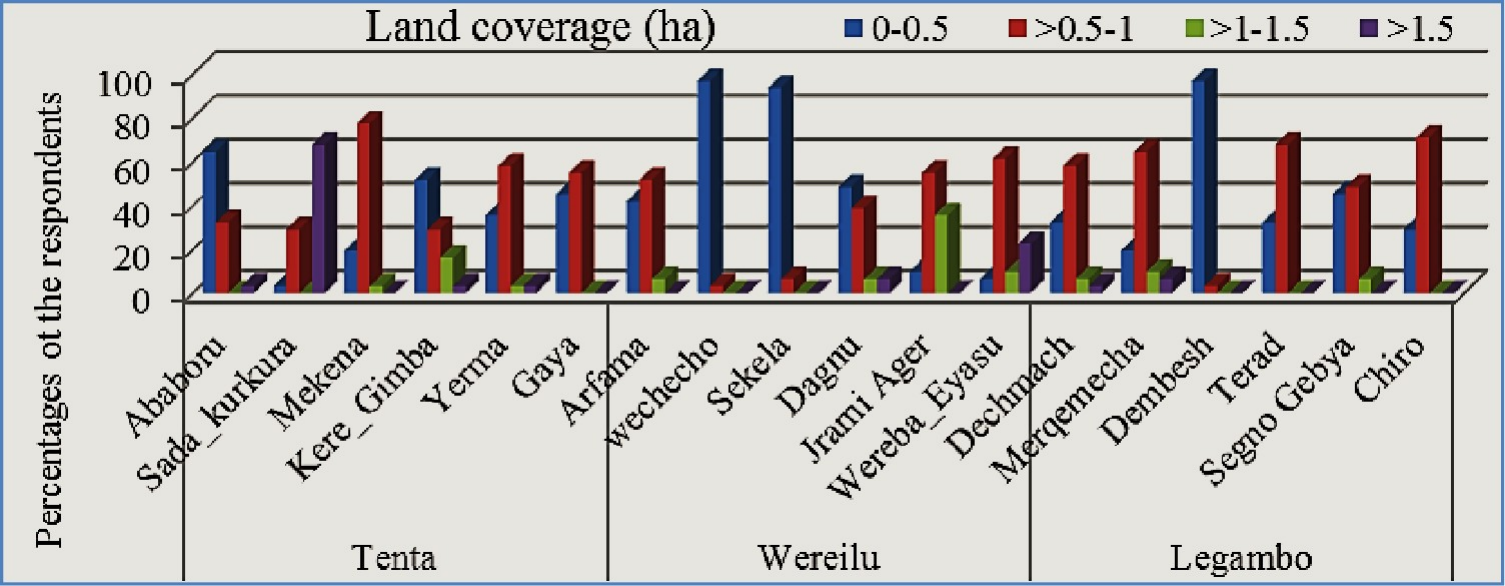
The land coverage by the barley landraces in each kebeles.

**Fig 2:**
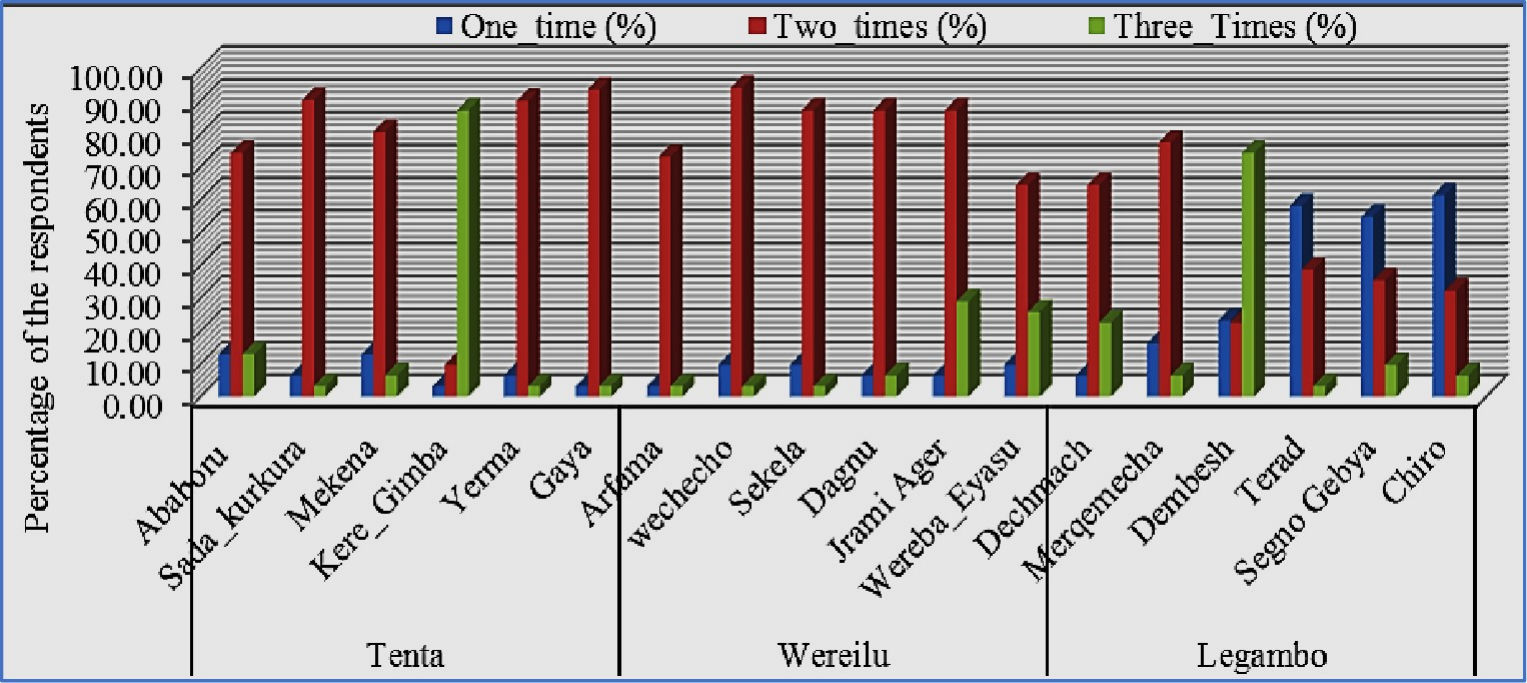
The ploughing frequency of the land before sowing.

**Fig 3:**
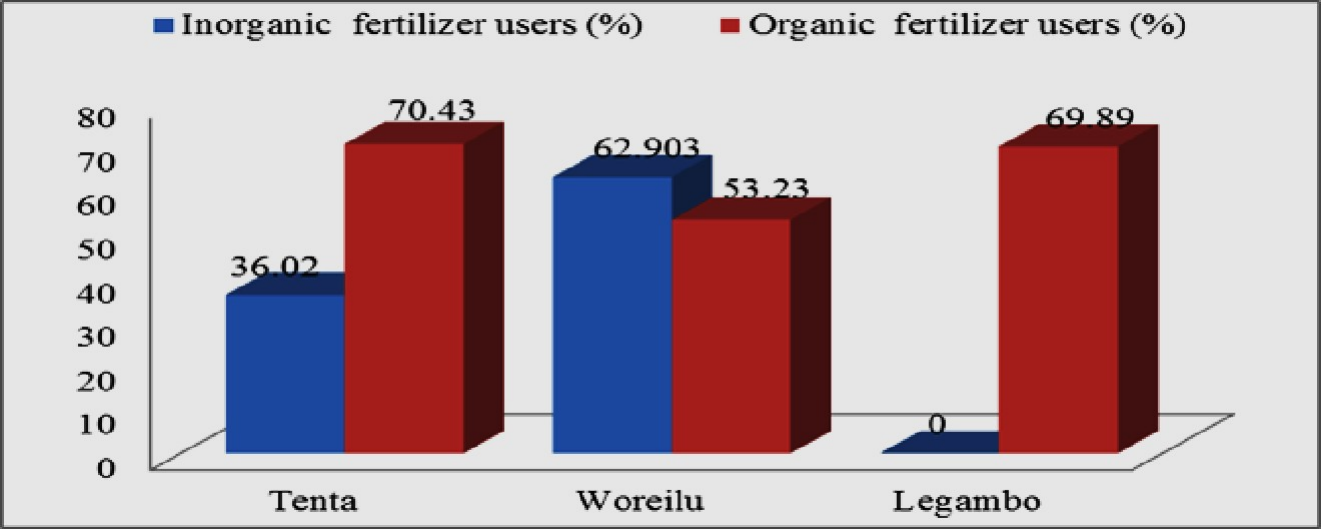
The three district fertilizer utilization and its application.

Most farmers had ≤1 ha barley; some had ≥1.5 ha of barley ((Fig 1). At farm, most farmers were plowing twice before sowing using horse (Fig 2). They are ploughing their land on October and second on January to February. Three times ploughing system was preferable and planted well nurture barley and given high yields. In the study area, ploughed by horse was common practice and this system was traditional, tiresome and required two persons. Biological fertilizer usages were well practice at Tenta, Legambo and Were’ilu, respectively. While, inorganic fertilizer usage was better at Wereilu, but none at Legambo (Fig 3). Pest management was better at Wereilu and hand weeding is a common system, but low at Legambo, and mowing by sickle, threshing by horse and store in Gotera were a shared practice. Result revealed that most farmers use broadcasting fertilizer and some of them use side dressing fertilizer. Practically, farmers proved that broadcast way of fertilizer is less effective than other methods.

#### Effect of weed and pest management in the local barley production

**Fig 4:**
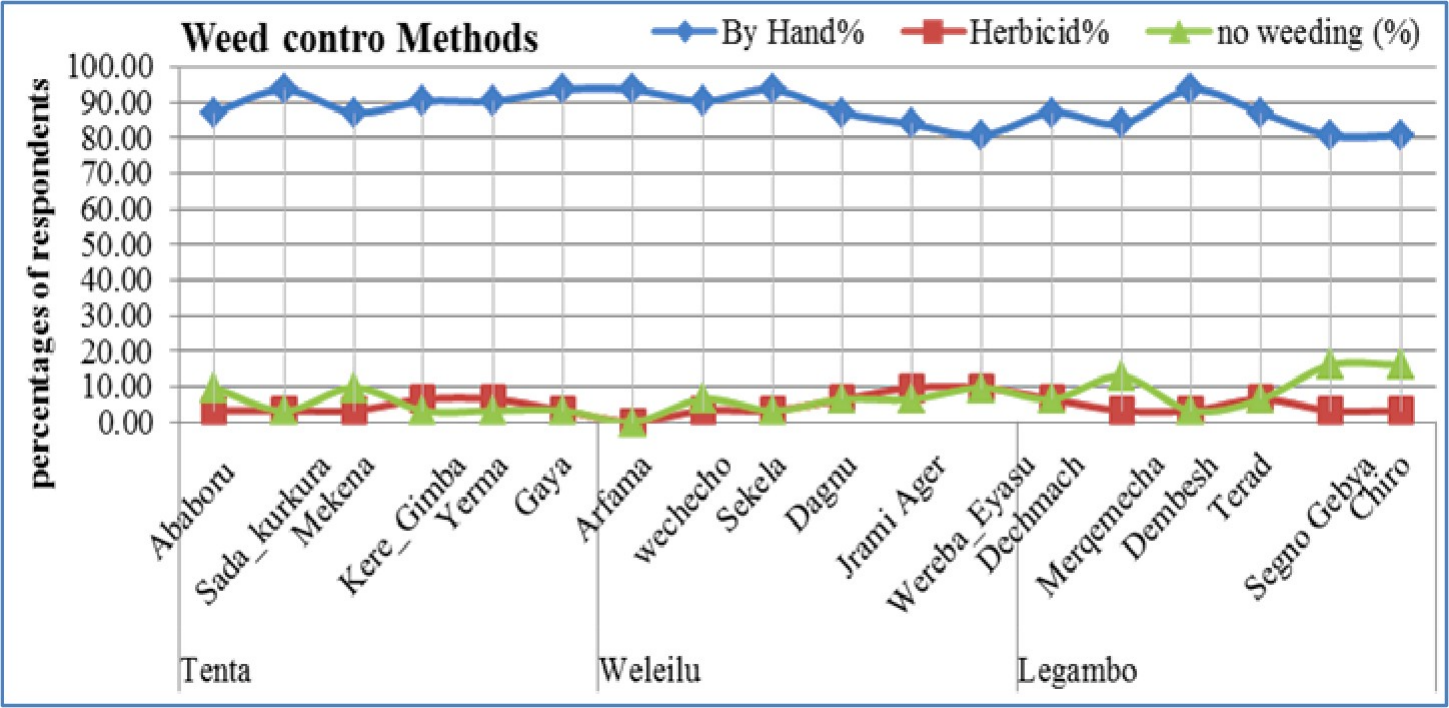
Weed controlled methods in the three districts and kebeles among districts.

As we proved that weed controlling were done in the farmers by hand, while in Legambo either a little weeding practice were done as compared to Tenta and Wereilu (Fig 4). Weed infestation is biotic factor that is responsible for the low barley grain yield. Generally, weeds reduce crop yields by competing for light, nutrients, water and carbon dioxide as well as interfering with harvesting [12]. Weeding frequency depends on the infestation level; two times of hand weeding is recommended at 25–30 days after sowing and 45–55 days after sowing [13]. In the insight of the farmers we proved that hand-weeding is to be the better weed controlling method and this was reflected in higher barley yields in the three districts. In the same way hand weeding enable to reducing the price for herbicides. Hand weeding system for barely production was recommended, for instance; [12] reported that hand weeding system increasing the grain yield. Pest management systems were not highly practiced enough, but farmers in Wereilu districts had better management habit as compared to farmers in Legambo and Tenta districts. Rats are the challenging factors that affect their local barley varieties and grains.

### Post-harvest management

**Fig 5:**
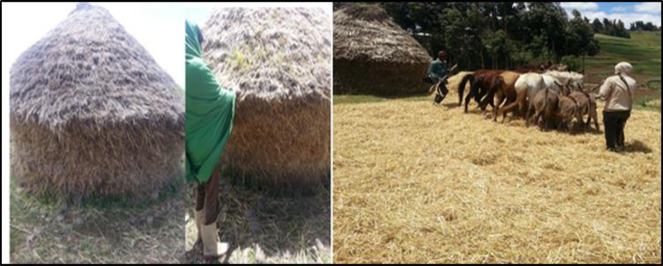
Traditional field storage and threshing practice of barley landraces and its side effect.

In these districts, 100% of the farmers were harvesting their crops using hand tools such as sickles. This result was in contrast with [14], and stated that only 10% of farmers in Southeast of Turkey did harvesting by hand. Mostly harvesting was done on June (known as summer), and they store at fields (3–4 months before threshing) (Fig 5). Therefore, barley was germinating and lost in the result of humidity, temperature and rodents. Threshing was done by horses and donkeys, and it was store under shelter on the floor and kasha/gotera/bags. This result was confirmed by [2], they proved that the outgoing and incoming of grain was stored in traditional containers called gotera. Rodents have been one of the biotic factors that cause damage both in the field and in storage. High levels of food insecurity continue in the belg dependent areas of Amhara region, because of 75% loss of belg harvesting. Monitoring report indicate that cases of acute malnutrition are widespread in some woredas including Legambo of South Wollo [15].

## Conclusions and recommendations

In this regard, the eight local barley varieties (Sene/Nech, Tegadime, Temezhi, Tikur, Belg, Ginbote, Traveler and Holker) were identified and of these six of them were evenly distributed in the three districts, but Temezh and Traveler unevenly distributed. We proved that, inorganic fertilizer usage was different in the three districts, but they shared similar organic fertilizer usage and application. As we seen in the field, the Agronomic and post-harvesting management system in three districts are almost related. Rodents, rainfalls were the common observed problems before and after threshing of their crops and no updated Machineries were available.

Therefore, to sustain the existence of the local barley varieties, different conservation management and new agronomic and post-harvesting technologies (Mechanical thresher’s machines, manually-operated or engine powered, tractor operated ploughing,) should be addressed.

## Supporting information

S1 Table List of landraces recorded in each district with Shannon diversity index and evenness. (DOCX)

## References

[1] Hailemichael S, Sopade P. Ethno botany, diverse food uses, claimed health benefits and implications on conservation of barley landraces in North Eastern Ethiopia highlands. Journal of Ethnobiology and Ethnomedicine, 2011; 7:19. 10.1186/1746-4269-7-19 21711566 PMC3148959

[2] Eticha F, Woldeyesus S, Heinrich G. On-farm Diversity and Characterization of Barley (*Hordeum vulgare* L.) Landraces in the Highlands of West Shewa, Ethiopia. Ethnobotany Research & Applications, 2010; 8:025–034.

[3] Gashaw G, Tura K. Review of Barley Value Chain Management in Ethiopia. Journal of Biology, Agriculture and Healthcare, 2015; 5.

[4] CSA (central statistical agency). Agricultural sample survey: area and production of major crops, Meher season. Vol. I. Addis Ababa, Ethiopia, 2018.

[5] Muluken B, Jemal E. Achievements in food barley breeding research in the early production systems of northwest, Ethiopia. In:Mulatu, B. and Grando, S. (eds). 2011. Review of Barley Research and Development in Ethiopia. Proceedings of the 2nd National Barley Research and Development Review Workshop.28-30 November 2006, HARC, Holetta, Ethiopia, 2011.

[6] Girma M. Genetic erosion of barley in North Shewa Zone of Oromiya Region, Ethiopia. International Journal of Biodiversity and Conservation, 2014; 6(3):280–289.

[7] Seid E, Eleni S, Faris H. Evaluation of genetic diversity in barley (*Hordeum vulgare* L.) from Wollo high land areas using agro-morphological traits and hordein. Afr. J. Biotechnol. 2015; 14(22):1886–1896.

[8] Simpson EH (1949).Measurement of diversity. Nature 163:688.

[9] Magurran AE. Ecological diversity and its measurements. Croom Helm, London,UK 1988. pp125.

[10] Tadesse D, Asres T. On farm diversity of barley landraces in North Western Ethiopia. Int. J. Biodivers. Conserv. 2019; 11(1):1–7.

[11] Abay F, Bjørnstad A, Smale M. Measuring on Farm Diversity and Determinants of Barley Diversity in Tigray, Northern Ethiopia. MEJS. 2009; 1 (2):44–66. 10.4314/mejs.v1i2.46048

[12] Tawaha AM, Turk MA, Maghaireh GA. Response of barley to herbicide versus mechanical weed control under semi‐arid conditions. Journal of agronomy and crop science, 2002; 188(2):106–112.

[13] Hassen A, Tafere M, Tolla M, Dagnew S, Ahmed A, Yihenew G, Molla D, Worku A. Capacity building for scaling up of evidence-based best practices in agricultural production in Ethiopia: Best Fit Practice Manual for Food Barley Production, 2015.

[14] FAO. FAO Statistics. Food and Agriculture Organization, Rome, Italy; 2004.

[15] WFP. Ethiopia Food Security Update: Famine Early Warning Systems Network and World Food Programme, 2009. www.fews.net/ethiopia.

