# Supplementary material for: Diversity, distribution, agronomic and post-harvest management of local barley (Hordeum vulgare L.) variety in South Wollo, Ethiopia
Source: PLoS One. 2021 May 6;16(5):e0250899. doi: 10.1371/journal.pone.0250899 (PMC8101768; doi:10.1371/journal.pone.0250899)
Supplement: S1 Table — (DOCX) [file pone.0250899.s001.docx]

**Supporting Information**

| Variety | | | | | Frequency | | | | | | |  |  |  |  |  |  |  |  |
| --- | --- | --- | --- | --- | --- | --- | --- | --- | --- | --- | --- | --- | --- | --- | --- | --- | --- | --- | --- |
|  |  |  |  |  | Tenta | Legambo | | | Wereilu | | |  |  |  |  |  |  |  |  |
| Sene/Nech | | | | | 91 | 95 | | | 100 | | |  |  |  |  |  |  |  |  |
| Tegadmie | | | | | 13 | 3 | | | 12 | | |  |  |  |  |  |  |  |  |
| Temezhi | | | | | 7 | 1 | | | 0 | | |  |  |  |  |  |  |  |  |
| Tikur | | | | | 29 | 73 | | | 18 | | |  |  |  |  |  |  |  |  |
| Belg | | | | | 21 | 11 | | | 9 | | |  |  |  |  |  |  |  |  |
| Ginbote | | | | | 22 | 11 | | | 53 | | |  |  |  |  |  |  |  |  |
| Holker | | | | | 1 | 2 | | | 1 | | |  |  |  |  |  |  |  |  |
| Traveler | | | | | 10 | 0 | | | 3 | | |  |  |  |  |  |  |  |  |
| Number of landraces | | | | | 8 | 7 | | | 7 | | |  |  |  |  |  |  |  |  |
| Total frequency | | | | | 194 | 196 | | | 196 | | |  |  |  |  |  |  |  |  |
| Shannon Diversity Index (H) | | | | | 1.607 | 1.179 | | | 1.32 | | |  |  |  |  |  |  |  |  |
| Hmax= lnS | | | | | 2.079 | 1.946 | | | 1.946 | | |  |  |  |  |  |  |  |  |
| E=H/Hmax | | | | | 0.773 | 0.606 | | | 0.678 | | |  |  |  |  |  |  |  |  |
|  | | | | | | | | | | | |  |  |  |  |  |  |  |  |
| Site | | Variety | Number | Pi | lnpi | | | pi*lnpi | H | | Hmax= lnS | | | | E=H/Hmax | | |  |  |
| Tenta | | Sene/Nech | 87 | 0.467742 | -0.7598386 | | | -0.35541 | 1.612029 | | 2.079441542 | | | | 0.775222 | | |  |  |
|  |  | Tegadmie | 12 | 0.064516 | -2.74084 | | | -0.17683 |  |  |  |  |  |  |  |  |  |  |  |
|  |  | Temezhi | 7 | 0.037634 | -3.2798365 | | | -0.12343 |  |  |  |  |  |  |  |  |  |  |  |
|  |  | Tikur | 28 | 0.150538 | -1.8935422 | | | -0.28505 |  |  |  |  |  |  |  |  |  |  |  |
|  |  | Belg | 20 | 0.107527 | -2.2300144 | | | -0.23979 |  |  |  |  |  |  |  |  |  |  |  |
|  |  | Ginbote | 21 | 0.112903 | -2.1812242 | | | -0.24627 |  |  |  |  |  |  |  |  |  |  |  |
|  |  | Holker | 1 | 0.005376 | -5.2257467 | | | -0.0281 |  |  |  |  |  |  |  |  |  |  |  |
|  |  | Traveler | 10 | 0.053763 | -2.9231616 | | | -0.15716 |  |  |  |  |  |  |  |  |  |  |  |
|  | | | | | | | | |  |  |  |  |  |  |  |  |  |  |  |
| Site | | Variety | No | Pi | lnpi | | | pi*lnpi | H | | | Hmax= lnS | | | | E=H/Hmax | | |  |
| Legambo | | Sene/Nech | 90 | 0.483871 | -0.72594 | | | -0.35126 | 1.179174 | | | 1.9459101 | | | | 0.605976 | | |  |
|  |  | Tegadmie | 2 | 0.010753 | -4.5326 | | | -0.04874 |  |  |  |  |  |  |  |  |  |  |  |
|  |  | Temezhi | 1 | 0.005376 | -5.22575 | | | -0.0281 |  |  |  |  |  |  |  |  |  |  |  |
|  |  | Tikur | 69 | 0.370968 | -0.99164 | | | -0.36787 |  |  |  |  |  |  |  |  |  |  |  |
|  |  | Belg | 11 | 0.05914 | -2.82785 | | | -0.16724 |  |  |  |  |  |  |  |  |  |  |  |
|  |  | Ginbote | 11 | 0.05914 | -2.82785 | | | -0.16724 |  |  |  |  |  |  |  |  |  |  |  |
|  |  | Holker | 2 | 0.010753 | -4.5326 | | | -0.04874 |  |  |  |  |  |  |  |  |  |  |  |
|  | | | | | | | | |  |  |  |  |  |  |  |  |  |  |  |
| Site | | Variety | No | Pi | lnpi | | | pi*lnpi | H | | | | | Hmax= lnS | | | E=H/Hmax | | |
| Wereilu | | Sene/Nech | 95 | 0.510753 | -0.6718698 | | | -0.34316 | 1.323425 | | | | | 1.945910149 | | | 0.680106 | | |
|  |  | Tegadmie | 11 | 0.05914 | -2.8278514 | | | -0.16724 |  |  |  |  |  |  |  |  |  |  |  |
|  |  | Tikur | 17 | 0.091398 | -2.3925333 | | | -0.21867 |  |  |  |  |  |  |  |  |  |  |  |
|  |  | Belg | 9 | 0.048387 | -3.0285221 | | | -0.14654 |  |  |  |  |  |  |  |  |  |  |  |
|  |  | Ginbote | 50 | 0.268817 | -1.3137237 | | | -0.35315 |  |  |  |  |  |  |  |  |  |  |  |
|  |  | Holker | 1 | 0.005376 | -5.2257467 | | | -0.0281 |  |  |  |  |  |  |  |  |  |  |  |
|  |  | Traveler | 3 | 0.016129 | -4.1271344 | | | -0.06657 |  |  |  |  |  |  |  |  |  |  |  |
